# The RAIS Device for Global Surgery: Using a Participatory Design Approach to Navigate the Translational Pathway to Clinical Use

**DOI:** 10.1109/JTEHM.2022.3177313

**Published:** 2022-05-23

**Authors:** M. Marriott Webb, P. Bridges, N. Aruparayil, C. Chugh, T. Beacon, T. Singh, S. S. Sawhney, L. Bains, R. Hall, D. Jayne, J. Gnanaraj, A. Mishra, P. R. Culmer

**Affiliations:** School of Mechanical EngineeringUniversity of Leeds 4468 Leeds LS2 9JT U.K.; Pd-m International Thirsk YO7 1DA U.K.; Leeds Institute of Medical Research, University of Leeds 4468 Leeds LS2 9JT U.K.; Department of SurgeryMaulana Azad Medical College 28862 New Delhi 110002 India; Medical Aid International Bedford MK43 8TW U.K.; XLO Ortho Life Systems New Delhi 110020 India; Department of Academic SurgeryUniversity of Leeds 4468 Leeds LS2 9JT U.K.; Department of Electronics and Instrumentation EngineeringKarunya University 121735 Coimbatore 641114 India

**Keywords:** global health, medical device design, participatory design, frugal engineering, surgical technology

## Abstract

Background: Over 5 billion people worldwide have no access to surgery worldwide, typically in low-resource settings, despite it being a primary life-saving treatment. Gas Insufflation-Less Laparoscopic Surgery (GILLS) can address this inequity, by improving current GILLS instrumentation to modern surgical standards. Objective: to develop and translate a new Retractor for Abdominal Insufflation-less Surgery (RAIS) into clinical use and thus provide a context-appropriate system to advance GILLS surgery. Methods: A collaborative multidisciplinary team from the UK and India was formed, embedding local clinical stakeholders and an industry partner in defining user and contextual needs. System development was based on a phased roadmap for ‘surgical device design in low resource settings’ and embedded participatory and frugal design principles in an iterative process supported by traditional medical device design methodologies. Each phase of development was evaluated by the stakeholder team through interactive workshops using cadaveric surgical simulations. A Commercialisation phase undertook Design to Manufacture and regulatory approval activities. Clinical validation was then conducted with rural surgeons performing GILLS procedures using the RAIS system. Semi-structured questionnaires and interviews were used to evaluate device performance. Results: A set of user needs and contextual requirements were defined and formalised. System development occurred across five iterations. Stakeholder participation was instrumental in converging on a design which met user requirements. A commercial RAIS system was then produced by an industry partner under Indian regulatory approval. This was successfully used in clinical validation to conduct 12 surgical procedures at two locations in rural India. Surgical feedback showed that the RAIS system provided a valuable and usable surgical instrument which was appropriate for use in low-resource contexts. Conclusions: Using a context-specific development approach with close engagement of stakeholders was crucial to develop the RAIS system for low-resource regions. The outcome is translation from global health need into a fully realized commercial instrument which can be used by surgeons in low-resource regions across India.

## I. Introduction

Over 5 billion people globally have no access to safe and affordable surgery; a shocking statistic compounded by the fact that the majority of those without surgical provision reside in Low and Middle-Income Countries (LMICs) [1], [2]. This predicament has worrying resonance because *accessible*, *affordable* and *safe surgery* is integral to effective healthcare delivery in LMICs, as highlighted by the World Health Organisation [2], [3]. Surgery is fundamental to solving key challenges in these regions. [4], from ensuring maternal and child health to enabling treatment of increasingly prevalent incidences of cancer, road traffic injuries and cardiovascular diseases [1], [3]. Yet nine out of ten people in LMICs cannot access surgery, and those who can, risk impoverishment due to expenditures on accessing surgery [1], [5]. This critical inequity in global healthcare is not a simple consequence of insufficient infrastructure or resource in LMICs, but also the slow development and adoption of context-appropriate surgical technologies (encompassing both technique and associated equipment) [1], [6].

**FIGURE 1.**
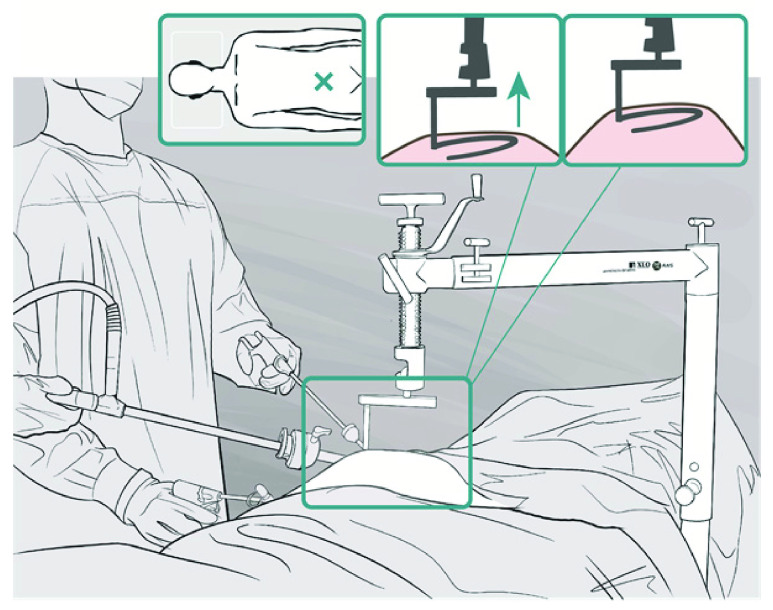
Gas Insufflation-Less Laparoscopic Surgery (GILLS) enables provision of key-hole surgery in low-resource environments, bringing access to life-saving surgical interventions in previously neglected communities.

Laparoscopic surgery brings several clinical benefits over traditional open surgical techniques which have the potential to reduce health burdens in LMICs, including faster recovery times and lower post-operative infection rates [7]. Laparoscopy involves ‘insufflating’ (inflating) the abdominal cavity using pressure-controlled Carbon dioxide (CO_2_) gas to form a pneumoperitoneum while the patient is under general anaesthetic and monitored by an anaesthetist. Unfortunately adoption of laparoscopy in LMICs has been slow, principally due to lack of these resources [8]. Gas Insufflation-Less Laparoscopic Surgery (GILLS) is an alternative form of laparoscopy, adopted by surgeons in rural areas of Northeast India, to address these challenges [9]. The technique, shown in Fig. 1, uses an abdominal-wall lift-device to physically raise the abdominal wall to create operative space. It makes laparoscopic surgery possible while the patient is under spinal anaesthesia and removes the need for general anaesthesia, a dedicated anaesthetist, CO2 gas and the associated specialised equipment. These efficiencies help overcome the resource barrier to the provision of surgery in low resource settings [10]. Consequently, GILLS has received increasing attention in studies which demonstrated its clinical safety and efficacy [11], recognition by surgical associations and has been acknowledged by the World Health Organisation (WHO) in their ‘*Compendium of technologies for low-resource settings*’ [12]. However, despite the potential of GILLS, global uptake has been slow. The factors responsible were explored in collaboration with clinical stakeholders in India [11], [13], [14]. Amongst a variety of factors, including the need for improved training and proctorship, usability studies identified that current GILLS instrumentation suffers poor functionality and robustness which act as barriers to its adoption in surgical use. Thus, there is a critical need for improved abdominal-wall lift-devices, designed to meet surgical needs in low resource settings, which forms the clinical target of this work.

To improve global access to medical technologies, the World Health Organisation (WHO) state that development of devices should focus on *‘4A’s’*: Affordability, Availability, Accessibility and Appropriateness [5]. An estimated 40% of the healthcare equipment in developing countries is not in use (compared to 1% in developed countries) [15], a sobering statistic that highlights the need for context-appropriate design and procurement [16]. To combat this issue, there is increasing recognition that new, more robust technologies must be designed specifically for low-resource contexts, with their performance carefully benchmarked against existing technologies [1], [17].

The broader process of designing medical devices, and specifically surgical devices, is an established field in which decades of industrial practice have led to the development of design process models which provide systematic frameworks to navigate all stages of medical device development, evaluation and commercialisation [18], [19]. While these models are an integral part of medical device design, they inherently focus on satisfying the requirements of high-resource healthcare systems (typically in High Income Countries (HICs)). However, the needs of low-resource healthcare providers in LMICs are typically very different to high-resource healthcare systems; they have limited funds for procurement and maintenance, fewer formally trained healthcare workers and underdeveloped supporting infrastructure [16], [17]. Consequently, the design process models instrumental to medical device development in HICs are not directly applicable to medical devices intended for low-resource healthcare providers. Thus, alternative strategies, targeted toward low-resource settings, are required to ensure context-appropriate surgical equipment which satisfy the WHO 4A recommendations [20].

The aim of this work was to develop an innovative abdominal-wall lift device which meets local stakeholder needs in low resource healthcare contexts. This paper reports the process of developing the Retractor for Abdominal Insufflation-less Surgery (RAIS), navigating the pathway from clinical-need to clinical-use using context-specific design approaches. Section II details the selection and development of appropriate methods, the outcomes of which are reported in Section III. The paper concludes in Sections IV and V, including a discussion on recommendations to guide other innovators seeking to develop new medical technologies for low resource settings. This builds on prior work which considers needs identification [21] to encompass the full process of design and translation through to clinical use.

## II. Methods

A growing body of research has sought to address the challenge of designing medical devices specifically for low-resource contexts which can address the WHO’s “4As” [5]. To date no holistic design frameworks exist (as found in medical device design for high-resource contexts) so a composite approach was developed by identifying and integrating best practice to span the development pathway [21]. Initial phases of development were guided by the ‘Roadmap for Design of Surgical Equipment for Safe Surgery Worldwide’ (referred herein as the ‘Design for Safe Surgery Roadmap’) [22] which places a necessary emphasis on understanding the nuances particular to low resource contexts for innovating appropriate prototype solutions [16].

To inform the activities conducted within this hybrid design framework, participatory and frugal design were selected as important ‘guiding principles’ due to their wide use in medical design for low resource settings and alignment with the project aims [9], [21]. Participatory design mandates close involvement of stakeholders in design activities (e.g. requirements-setting or design verification) founded on the ‘ideal’ of co-creation: ‘to equitably recognise the role of all stakeholders as innovators in all stages of the design process’ [23], [24]. Frugal design aims to find elegant design solutions which use less resource while achieving comparable performance by promoting solutions which avoid extraneous features and focus on key needs [25], [26]. The consequent outcomes are recognized to support disruptive improvements in global healthcare [27], [28].

**FIGURE 2.**
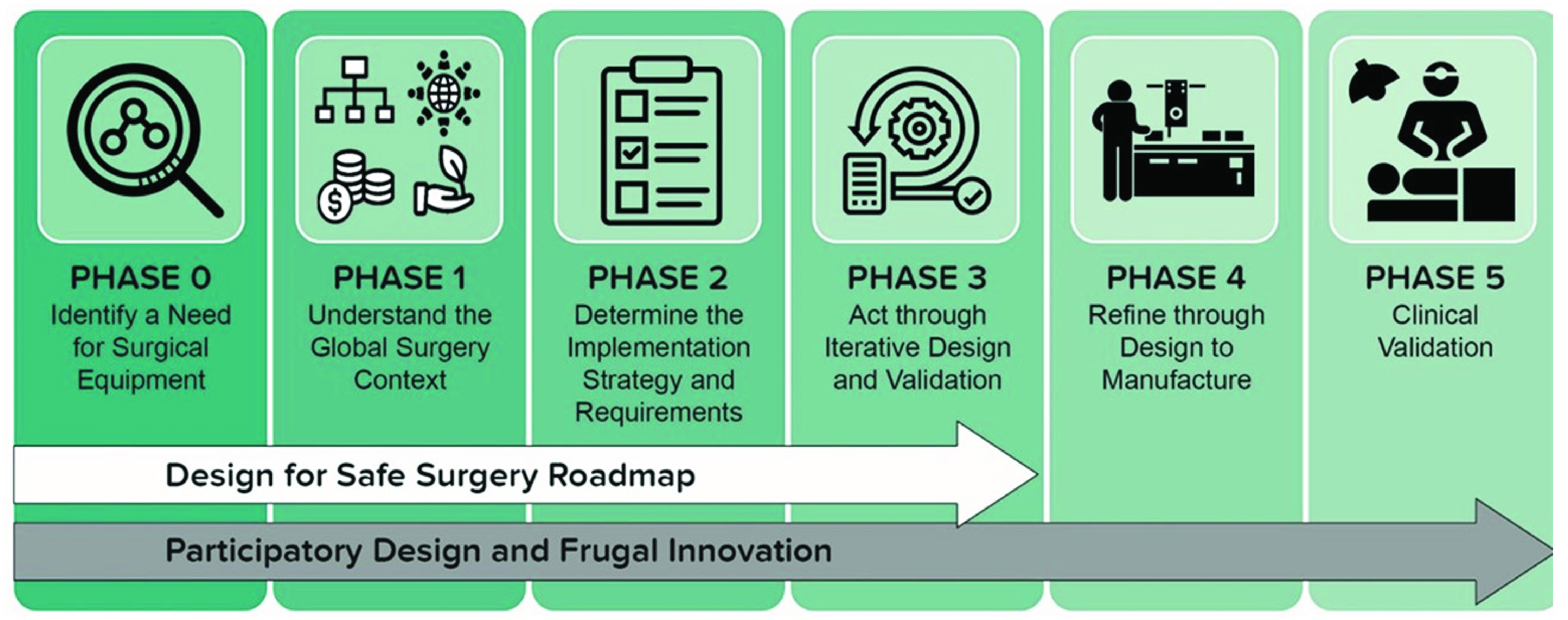
The translational pathway followed during development of the RAIS system, from clinical need to clinical validation.

The following sections describe the implementation of this approach as a series of interconnected activities aligned to phases defined in the ‘Design for Safe Surgery Roadmap’ and extended beyond to encompass commercialisation and clinical validation, as shown in Fig. 2.

### A. Phase 0-2: Essential Needs & Requirements

The need identification process is detailed in [21] and summarized here for context. The Design for Safe Surgery Roadmap recommends identifying needs through scientific research, Non-Governmental Organizations and local stakeholders [22]. The NIHR Global Health Research Group in Surgical Technologies (GHRG-ST) collaborated with surgical associations in India to identify and engage local surgical stakeholders working in rural surgery.

Research literature was used to define broad topic areas from which detailed primary research was conducted with stakeholders [21]. To obtain maximum input from the clinical stakeholders, the majority of research activities were conducted in India. The GHRG-ST observed GILLS procedures at rural surgery training camps and then conducted semi-structured interviews with stakeholders to capture limitations, needs and local contextual factors.

These data were refined to establish a set of User Requirements. Discussion with clinical stakeholders was used to inform the minimum and ideal device functionality necessary to perform safe surgery in GILLS. The process emphasized a frugal design approach but allowed for additional functionality if it could support wider-use in different environments. The outcome from this phase was formalized in a P-Diagram, a format widely used in Robust Design to capture essential details of a system [29].

### B. Phase 3: Concept Development and Validation

Phase 3 of the roadmap comprises the activities necessary to move from system requirements and definition into designs and physical prototypes. A Waterfall Design Process Model [30], [31] was selected to structure these activities which ensures that the nuanced low-resource-specific design inputs influence the entire development process [32]. The model takes early conceptual designs and progresses through prototypes of increasing fidelity. In this implementation, each design iteration included a verification step with stakeholders local to the design context using a variety of modalities including in-person meetings, video-conferencing and email exchanges. This was included to enable stakeholders to evaluate designs, contribute ideas and steer design decisions throughout the process. Validation activities were conducted at two key milestones; 1) the first fully-functional prototype and 2) the final prototype prior to commercialisation.

#### 1) Concept Development: Validation Activity 1

The first validation step evaluated the performance of the first high-fidelity RAIS prototype device, manufactured in a form suitable to perform simulated laparoscopic procedures using cadaveric models. This represents an important milestone in the development cycle to assess how effectively the Essential Needs and Requirements have been captured in a physical form.

The validation activity was designed as a mixed-method study, combining qualitative interview and semi-structured questionnaire feedback, to be conducted in a cadaveric surgical workshop at a large rural surgery conference in India. The recruitment target was between 12-20 participants attending the conference to facilitate input from a broad range of clinical stakeholders with experience in rural surgery. Ethical approval was granted in India (Martin Luther Christian University, Shillong, India. Ref: VI/I(8)/UREC/EA/272/2015-6111) and UK (University of Leeds, Yorkshire, UK. Ref: MREC 19-029).

Each participant in the study received an initial briefing about the RAIS system and the purpose of the workshop. They were then asked to complete two tasks; 1) assembling the RAIS prototype from its component parts and configuring it on a surgical operating table and 2) performing a simulated diagnostic laparoscopy with a cadaver using the assembled prototype. The cadaver was an adult male preserved using a ‘soft-fix’ technique such that the soft tissues remain compliant and suitable for laparoscopic simulation (e.g. retraction and manipulation of tissues).

After completing this process, each participant was asked to complete a questionnaire in conjunction with a semi-structured interview. The questionnaire had two components, firstly a NASA Task Load Index (TLX) usability study to objectively evaluate the device’s usability during assembly and a surgical procedure, secondly a questionnaire to rate how the RAIS device suits aspects of surgical use in low resource settings. The NASA TLX consists of six criteria using a 1-21 scale and has been used extensively in medical device development and is more comprehensive when compared to other workload analysis techniques (e.g. capturing aspects including frustration and performance) [33]. The subsequent interview was used to elucidate topics highlighted in the questionnaire. The questionnaire and participant instructions are in Supplementary Materials B)

#### 2) Concept Development: Validation Activity 2

The ambition of the second validation step was to evaluate the final iteration of RAIS with the clinical stakeholders to ensure it could progress to the commercialisation phase. The aim was to again use a cadaveric surgical workshop to provide a high-quality surgical experience.

This phase of the project coincided with the start of the COVID-19 pandemic and consequently travel and supply-chain restrictions were severely restricted and prohibited direct involvement of the key clinical stakeholders. Instead, a hybrid option was designed in which the activity was conducted in the UK by a surgeon experienced in GILLS, with clinical stakeholders from India participating remotely via a secure teleconferencing video-link. The video-link was configured to provide intraoperative and external viewpoints (a fixed view of the operating table setup and a roving view). This aimed to provide the remote stakeholders with a high-quality ‘immersive’ experience of the activity in which they could direct movement of the cameras and collaborate on the surgical process with the local (UK) surgeon. Similar to the first cadaveric workshop, a soft-fix Thiel cadaver was used in the study to provide a representative surgical experience.

After the surgical session each participant was provided with a recording of the session for analysis and asked to complete an electronic semi-structured questionnaire to capture feedback on the perceived performance and functionality of RAIS during the activity. Thematic analysis was used to identify pertinent topics from these data. Ethical approval for this activity was granted by University of Leeds (Ref: MEEC 20-023).

### C. Phase 4-5: Manufacture and Commercialisation

This phase of work entailed identification of an appropriate partner to undertake manufacture of the RAIS system and to market the system for commercial availability. This encompasses associated Design to Manufacture and regulatory work necessary to transform a prototype system into a commercial RAIS system.

The team first identified a range of in-country medical device manufacturers with the capacity to market and distribute the final system. Using an in-country partner provides a solution focused on long-term sustainability; helping mitigate global supply-chain issues and embedding expert knowledge of local regulatory requirements and processes [34]. Under NDA, a project representative met with the prospective manufacturers to discuss an overview of the RAIS system spanning the clinical need, overarching ethos of the project (to develop an affordable and sustainable device for a small emergent market) and key areas of manufacturing complexity. Manufacturers were subsequently scored against a weighted set of key qualities to enable objective selection by the highest total score.

After selection, the design team worked closely with the manufacturer to adapt and refine the RAIS prototype design for mass production. This process was conducted during the onset of the COVID-19 pandemic which precluded in-person meetings, instead moving to bi-weekly online meetings between the design team, manufacturers and local stakeholders. To ensure detailed consideration of the system, the online meetings were structured such that a first set considered the overall system and functionality, before moving onto a focused set of meetings, each of which considered a specific section of the system (e.g. a clamp assembly), clarifying or revising the design as necessary, with input from the whole team. The phase culminated with the manufacturer producing a commercial-prototype of the RAIS system based on these revised designs. This system was evaluated in a cadaveric study, performed remotely via videoconferencing, with feedback collected from clinical champions and stakeholder participants via a questionnaire.

A last design iteration was conducted to address feedback and finalise a design for the commercial RAIS system. The system and clinical use was evaluated by the manufacturer’s regulatory team to determine the device class and regulatory requirements for clinical use within India [35]. Documentation and supporting evidence was developed in collaboration with the design team. The manufacturer then filed a submission to the regulatory body (Ministry of Home Affairs, Indian Government), for a regulatory test license.

### D. Clinical Validation of RAIS

The final aspect of development was ‘completing the loop’ by conducting clinical validation of the RAIS device with the surgical stakeholders. This was led by clinical champions who supported a cohort of rural surgeons in conducting GILLS procedures using the RAIS device to generate informed feedback on performance of the system.

To address the challenge of rural surgeons working in disparate and remote locations, the team arranged GILLS training workshops at two surgical sites in rural India; JSS Medical College (JSSMC, Karnataka, India) and Martin Luther Christian University (MLCU, Shillong, India). Each workshop ran over two-days: day one involved familiarisation through general education in laparoscopy and GILLS, with specific demonstration and training on the RAIS device, day two involved participants conducting surgeries using RAIS under supervision of the clinical team.

Approvals were obtained to conduct the workshops and collect study-specific information from the participants at each location (Martin Luther Christian University, Shillong, India. Ref: VI/I(8)/UREC/EA/272/2015-6111). Surgical procedures were conducted using the RAIS system under regulatory approval as part of the standard instrumentation set for pre-existing patient cases. Surgical participants were recruited by the clinical team based on their knowledge of the rural surgery network, identifying individuals within practical travel distance of each workshop location. Each gave informed consent to participate and provide feedback.

Feedback was obtained from each participant at the end of each workshop through semi-structured questionnaires and interviews, designed to capture information on RAIS (setup, cleaning, sterilization and maintenance), familiarisation to the device, achieving an intra-operative view and overall satisfaction, as shown in Supplementary Part C.

## III. Results

The RAIS system was successfully developed according to the overall plan detailed in Section II, to span Phases 0-5 as shown in Fig. 5. Some adaptation was required due to travel constraints imposed by the COVID-19 pandemic during Phase 4. Outcomes are detailed for each phase below.

**FIGURE 3.**
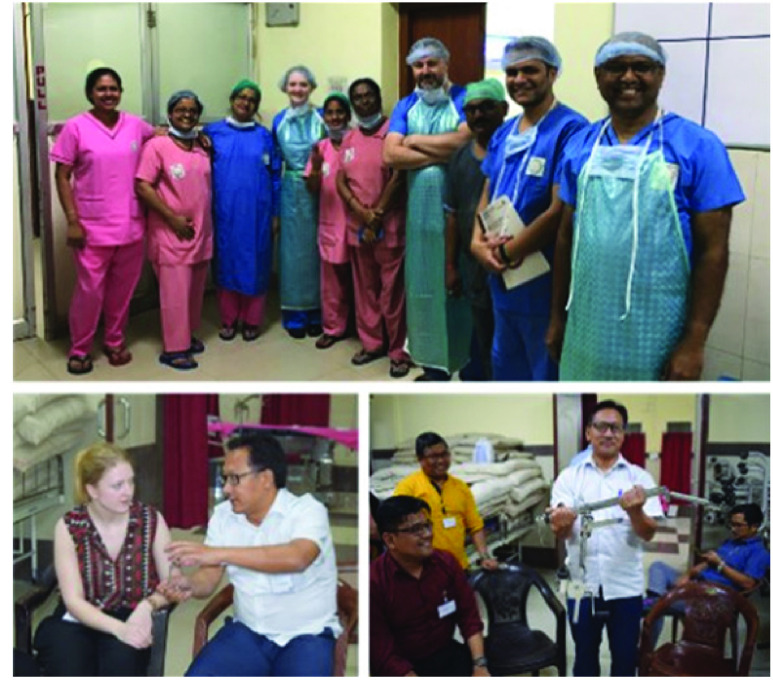
Identification of user needs and the environmental context were approached using the principles of Participatory Design, involving close collaboration between designers and rural surgery practitioners.

**FIGURE 4.**
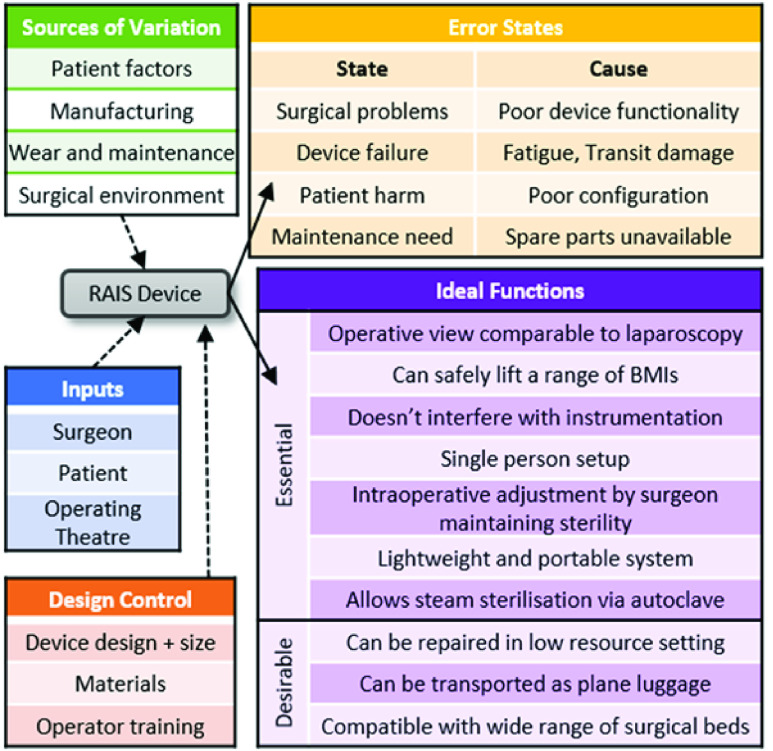
A high-level P-Diagram for the RAIS system defining the user needs, requirements, and environmental context, together with error states.

**FIGURE 5.**
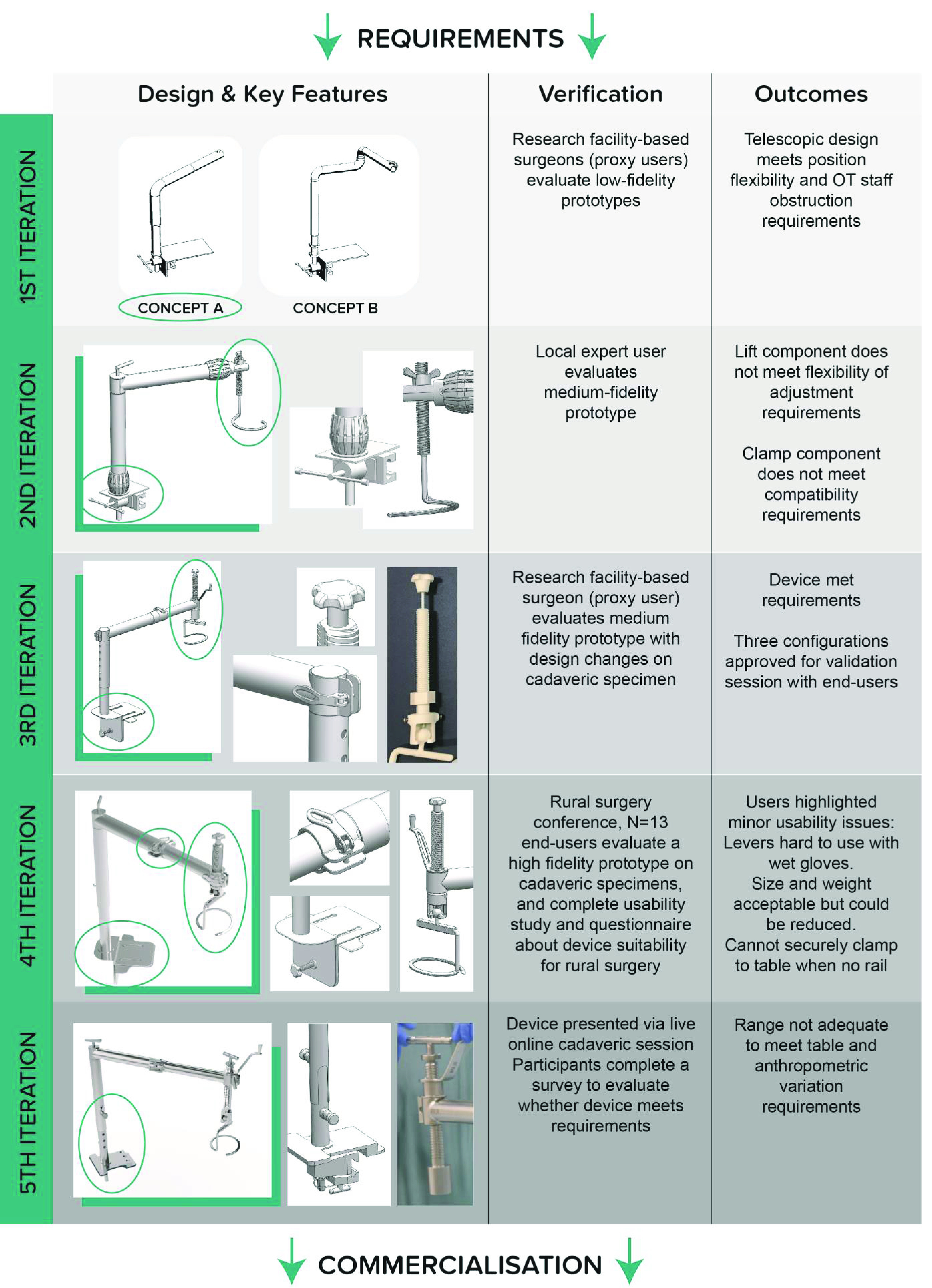
A summary of the process used in the iterative development, validation and verification of the RAIS system.

### A. Essential Needs and Requirements

To complete Phases 0-2 and develop clinical and contextual requirements required close collaboration with the stakeholders and clinical champions, as shown in Fig. 3. The process is described in detail in [21] and summarized here for context. A P-Diagram was used to formalize and delineate this information into incoming information; *Inputs*, *Design Control*, *Sources of Variation* and the associated outcomes as *Ideal Functions* and *Error States*. Fig. 4 shows an illustrative high-level P-Diagram relating to the RAIS system. Key *Inputs* are the surgeon, patient and operative environment. *Sources of Variation* defines aspects of variability which the device must accommodate, encompassing factors such as the patient (e.g. BMI) and device (e.g. wear). *Design Control* shows what can be regulated through careful development of the system which again includes device specifics (e.g. materials) together with usage factors (e.g. training). From these, the *Ideal Functions* then define the core attributes of the system which must span the full use-cycle; from transportation and setup through surgical performance to cleaning and maintenance. In tandem, Error States encompasses factors from manufacture through device performance (e.g. mechanical failure) to long-term maintain of the system.

### B. RAIS Concept Development and Validation

**FIGURE 6.**
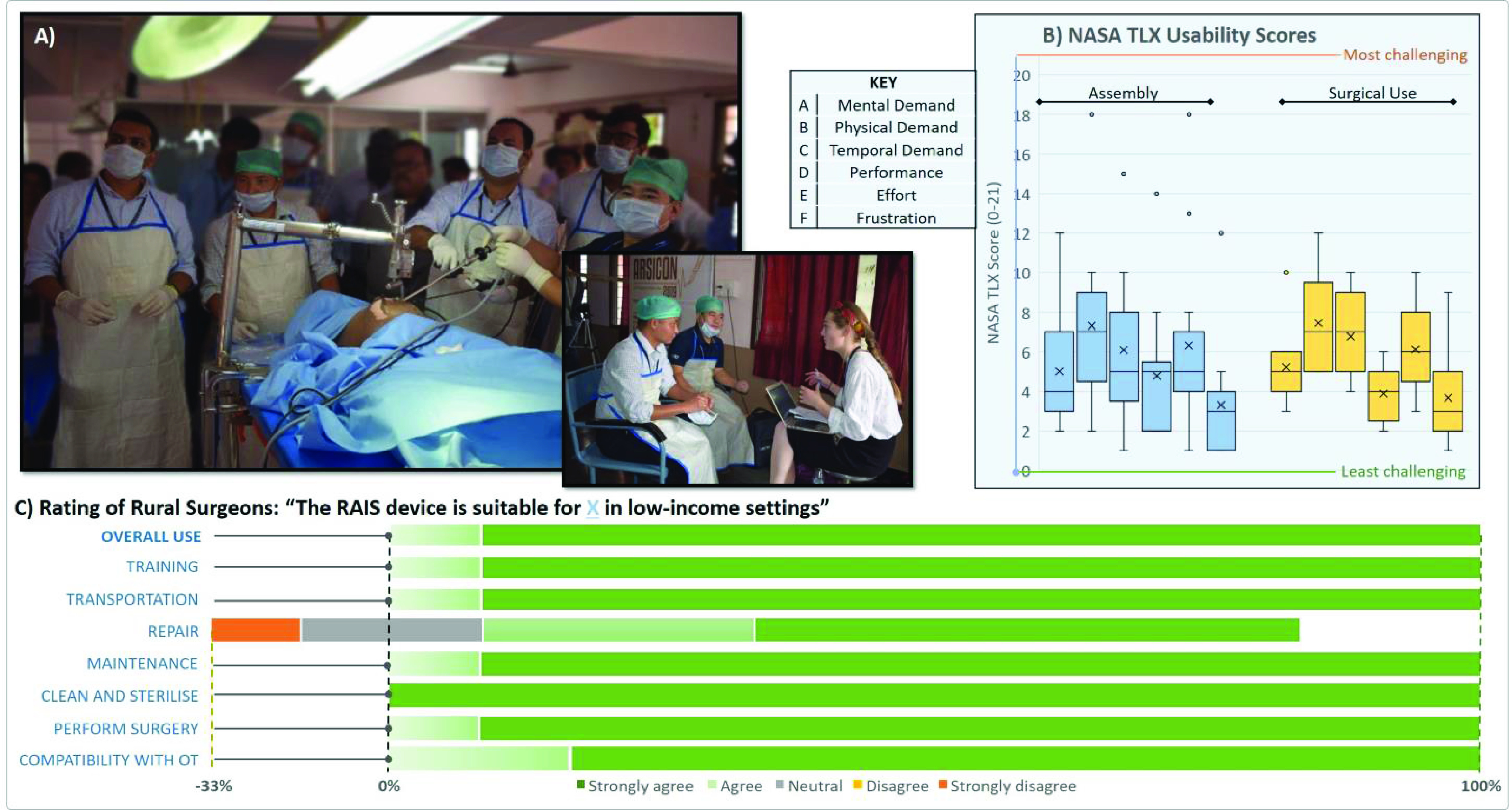
Stakeholder evaluation of the RAIS system during the development process with a) surgical simulation with stakeholders using a cadaveric model b) NASA TLX usability scores from the stakeholder cohort (0-21 scale) c) outcomes of the questionnaire rating functionality of the RAIS device by the surgical cohort.

To complete Phase 3 and achieve the user specification determined in previous phase, the team conducted five complete design iterations, as summarized in Fig. 5. The stakeholders were an integral part of the process, facilitating convergence towards a final design:

Iteration 1: The development process was initiated with the Context and Requirements information (see Fig. 4) from which the design team developed a set of three conceptual designs. Each design represented a different approach to achieving the core *Ideal Functions*, focusing on kinematic mechanism which could be configured to achieve the necessary movements and workspace of the system. These were realized as 3D CAD animations and low-fidelity physical prototypes (e.g. plastic tubing and stock joints) for initial evaluation with stakeholders in a video-conferenced workshop session with a mock-up surgical bed. Stakeholder feedback was influential in guiding the design direction; the design team had favoured a planar two-link arm concept (like a SCARA robot) but stakeholders identified that this would obstruct surgical practice and unanimously preferred an alternative concept using tubular telescopic sections.
Iteration 2: The telescopic design was refined, focusing on selection of representative components and sizing (e.g. tube dimensions) and providing functionality for the surgeon to control lift. A medium fidelity prototype was produced of the new design, employing 3D printed parts to represent key components such as the lift assembly. This prototype was robust for unloaded demonstration and was evaluated by visiting stakeholders in a physical workshop with a model abdominal wall and operating table. This in-person evaluation enabled a detailed exploration of the design and confirmed the value of the telescopic design, but highlighted that the lift assembly did not provide sufficient movement flexibility for surgery. Testing also revealed the clamp assembly, linking to the operating table, was critical to achieving appropriate performance; remaining stable under high load while accommodating a range of surgical rail sizes (and states of repair) found in low resource settings.
Iteration 3: focused on verifying the system in a more representative surgical environment. Revisions were made to the design of the lift and clamp assemblies to improve their functionality and robustness to *Source of Variation*. A fully functional prototype was manufactured to be capable of partial load-bearing (using a combination of machined metal and 3D printed parts to satisfy cost and time constraints). A cadaveric study was conducted, with a GILLS experienced surgical stakeholder conducting a simulated procedure (diagnostic laparoscopic sweep). The activity verified that 3D printed components (e.g. of the lift assembly) were appropriate in function with some minor changes in detail.
Iteration 4 – Development Validation Activity 1: entailed evaluation of a prototype RAIS system (see Methods B.1) and revision of the system based on the outcomes. A high-fidelity fully functional prototype of RAIS was manufactured, based on the design verified in Iteration 3, as shown in Fig. 5. This utilised representative medical-grade stainless-steel to ensure load-bearing capability.
  The planned surgical evaluation workshop was conducted as part of an Indian rural surgery conference (ARSICON2019, Nijalingappa Medical College, Bagalkot, India: The 27th Annual National Conference of Association of Rural Surgeons of India *ARSI* 2019). A cohort of 13 stakeholders, each working in low-resource hospital settings within India, were recruited. Informed, written consent was obtained from each participant. The majority of participants were surgeons with experience in GILLS from Northeast India (N = 9). Other participants (N = 4) included a General Practitioner, a Registered Nurse and two surgeons without prior experience in GILLS. All participants were able to complete assembly and configuration of the device. The participants with prior GILLS experience all successfully performed a diagnostic laparoscopy procedure using RAIS with the cadaveric surgical simulation.
  Fig. 6 shows a summary of the responses from the workshop. For assembly of the RAIS device, participant responses have a consistently low mean across the six subscales of the NASA TLX, showing low cognitive, physical and temporal aspects with good performance. This signifies that overall, the RAIS device was convenient to assemble in a timely fashion without significant effort or frustration. The outliers in these data correspond to participants without prior surgical experience. This may therefore be reflective of the learning curve required to operate surgical equipment in general, rather than RAIS specifically. For surgical use, participants reported low averages across all the NASA TLX subscales, indicating good usability and operational performance in general. The physical subscale was rated highest for both assembly and usage and consequently explored in the interview. Responses from the cohort revealed some concerns with the shape of the RAIS device adjustment fixtures which were judged challenging to manipulate while wearing surgical gloves, a subtle but important usability aspect to identify. The questionnaire, shown in Fig 6c, reveals that participants rated the core Ideal Functions of the RAIS device (see Fig. 4) to be well-suited for low-resource settings. The topic of repair was the sole exception to the positive feedback. Exploration of this aspect during the interview sessions revealed that some participants were concerned about a lack of engineering expertise local to their facilities having experience or capabilities to work with medical-grade stainless steels and/or medical devices. They would not feel comfortable trusting local mechanics with a surgical device that they could not easily replace, and were concerned about the potential delays or disruption in calling for external support from a supplier or ‘qualified’ mechanic.
Iteration 5: finalised development of RAIS prior to commercialisation. The design was modified to address the concerns identified in Iteration 4. Key design changes were focused on usability refinement (e.g. making adjustment mechanisms consistent across the system), and increasing robustness (e.g. reinforcement of the clamping assembly) and long-term maintenance (e.g. simplifying fixtures) to address concerns related to the need and efficacy of repair.

*Development Validation Activity 2:*

A revised high-fidelity prototype was successfully produced for evaluation in the UK-based cadaveric study. Four stakeholders from India were able to effectively participate via the video-conferencing facility. Each participant was a surgeon skilled in GILLS and working in low resource settings. This cohort guided the actions of the local lead surgeon through continuous dialogue. Thematic analysis of the questionnaire responses was conducted with respect to the device’s Ideal Functions. Assembly and configuration were deemed acceptable, with the caveat that first-hand experience would be necessary to be fully confident. Performance in surgical use was considered good, particularly in the ability to easily position the system, considered an improvement over previous iterations. However it was noted that the system lacked sufficient range in height adjustment. This aspect was addressed through a minor modification (extending the length of a component). Cleaning and sterilization was praised, particularly for the compatibility with compact autoclaves. Similarly, the prospective ease of transportation was commended due to the custom packing case with segmented compartments for each component part. Overall, despite the necessary limitations of this activity (i.e. participants were a small cohort of clinical champions associated with the project and interacting remotely), it provided sufficient evidence from experts, particularly given the context of a world pandemic, to recommend that this iteration of RAIS satisfied the *Ideal Functions*, mitigated against key *Error States*, and could thus progress to Commercialisation.

### C. Commercialisation of the RAIS System

**FIGURE 7.**
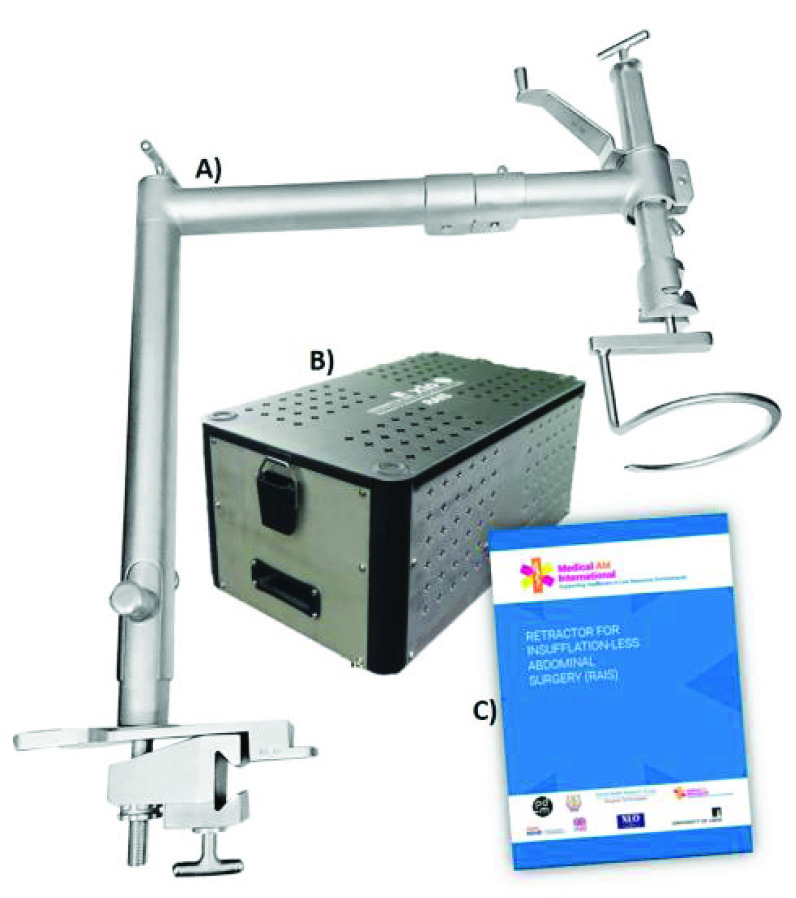
The commercial version of RAIS a) assembled b) in custom casing for transportation and sterilization c) Instructions for use.

A manufacturing partner was identified based on their combined technical manufacturing capabilities, established reputation, regulatory knowledge, quality of communication, and capabilities in distribution and marketing. The Design for Manufacture (DfM) process had to be conducted at distance due to COVID-19 restrictions, but these constraints were mitigated through regular online communication and evaluation of test pieces produced by the manufacturing partner. A final commercial version of RAIS was thus produced, as shown in Fig. 7. Major outcomes from the DfM process were:

• Selection of appropriate medical grade materials and tube-stock for the system (ISO 7153-1:2016)
• Revision of the lift-assembly for manufacture and robustness (e.g. optimizing thread design and consolidating multi-part components into single-piece to remove unnecessary joins
• Application of scratch-resistant shot-blast finish
• Optimisation of weld, chamfer and tolerancing geometry for ease of fabrication and cleaning
• Laser-etched labels and part numbers to guide usage (e.g. extension limits)

In conjunction, the manufacturing partner developed a custom casing system for the RAIS device to provide a means of safe transportation (to avoid component damage and loss), and to provide a consistent means for steam sterilization with components held on auto-clavable trays).

After the initial DfM activity, a sample system was produced for evaluation by local clinical stakeholders in conjunction with the UK design team. Minor amendments were specified on component geometry relating to the surgical lift ring which requires a specific spiral configuration to ensure appropriate surgical performance, after which the stakeholder team ‘signed off’ for commercial production.

Classification and regulation of surgical devices is controlled by the Health Ministry of India (part of the Indian Government) [36], [37]. The manufacturer’s regulatory team identified that the RAIS system is considered as a Class-A device (Low-risk surgical instrument) and worked with the Design Team to compile the requisite documentation. This encompassed documents including a risk-register, device description and verification of key performance claims (e.g. device load capabilities meet requirements in laboratory tests [38], [39] and surgical performance using cadaveric studies). After this process the manufacturer was issued with a Test License for the RAIS system, enabling clinical evaluation for a limited duration prior to award of a full license.

### D. Clinical Validation of RAIS

The clinical evaluation workshops were conducted as planned with a total of 30 stakeholders participating across 12 surgical procedures comprising appendectomy (4), hysterectomy (2) and cholecystectomy (6). No adverse avents were recorded as a result of the RAIS device and all surgeries were completed succesfully.

**FIGURE 8.**
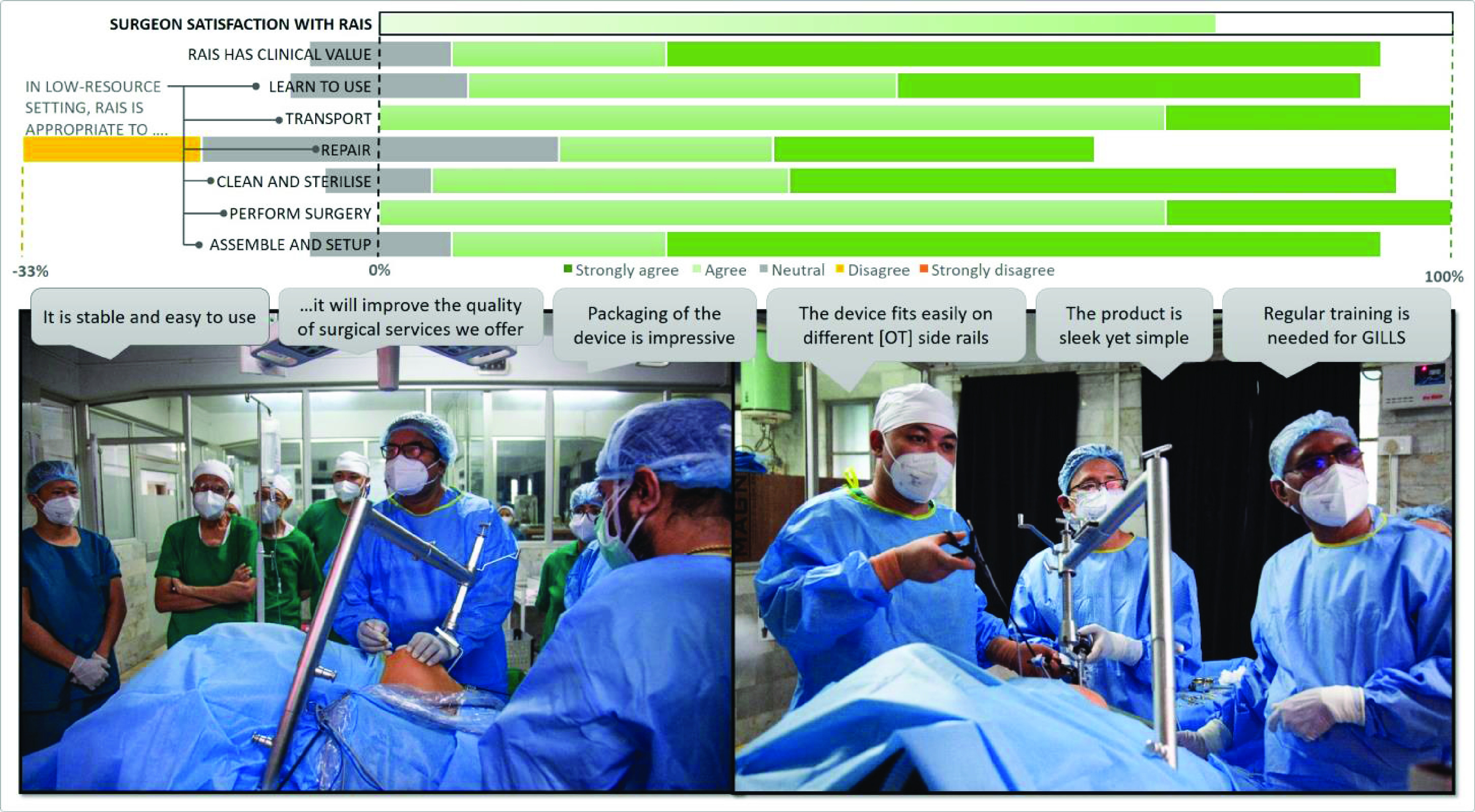
Clinical validation of the RAIS system, conducted in Surgical Training workshops, and a summary of their feedback.

Fig. 8 shows a representative surgical scenario of the RAIS device from the workshop, together with a summary of stakeholder feedback from the questionnaire, which was completed by all participants. Complete data are included as Supplementary materials. Foremost, the results demonstrate that RAIS was considered suitable and valuable as a tool to support gasless surgery in low-resource surgical settings, meeting the primary goal of this project. This aligns with the high Surgeon Satisfaction score which reflects practical experience of using the device. Feedback was also positive with respect to key stakeholder needs; RAIS was considered convenient to setup in the operating theatre, could be readily cleaned and sterilised and transported within and between rural surgical sites. The only negative aspects reported were with respect to repair of the device, with some participants concerned that low-resource facilities would not have the requisite facilities to address faults that may occur. This outcome highlights the need for both a robust system (thus minimising the need for repair) together with provision of manufacturer-supported repair options and spare parts.

## IV. Discussion

**The ambition** to develop the RAIS system into a commercially available, and clinical valuable, surgical system, necessitated a robust approach that could effectively embed a wide range of expertise throughout the translational pathway. The principle of Participatory Design was instrumental in this respect. It required significant time and resource to implement effectively, with activities spanning identification of stakeholders prior to starting the project through to regular interaction to maintain engagement throughout the project. However, this investment proved invaluable in achieving these ambitions and ensuring longer-term viability for the system, resonating with similar findings in the literature [40], [41]. From the outset, it underpinned an ethos of embedding stakeholders within the team, such that it was a case of ‘designing with’, rather than ‘designing for’ this group. This has profound implications on design methods in which it influenced the selection of the over-arching iterative development approach and then guided its implementation, from conceptual design to validation of outcomes. Like others conducting this work, it was found that including multiple representatives from different disciplines helped ensure a robust design process and avoid over-reliance on individual opinion which may not be representative [42], [43]. This highlights the importance of identifying a representative team of stakeholders with whom to work, and having the contacts and mechanisms to do so. Engaging with established networks greatly facilitated this process, in particular working with representative surgical associations (e.g. the Association of Rural Surgeons of India and NIHR Global Health Research Group) opened links to stakeholders in remote regions with invaluable expertise in rural surgery who may otherwise have been excluded.

*The Over-Arching Approach:* used to navigate development and clinical translation of the RAIS device was necessarily bespoke, since no over-arching framework exists for the specific needs of developing medical devices for low resource settings, in stark contrast to the multitude which target devices for high resource settings [44]. Here, a composite framework was formed by augmenting the Design for Safe Surgery Roadmap [21] with approaches selected from the literature which aligned with the principles of Participatory Design. In particular, the iterative approach of the ‘Waterfall’ method (see Fig. 5) actively promoted regular interaction with stakeholders in design, validation and decision making activities throughout development [30]. The result was an agile process supporting rapid refinement of the design while still remaining flexible to design changes (e.g. adaptation of fixtures in the 4^th^ Iteration) which were pivotal in achieving a final prototype system which met with end-user approval. While it is convenient to describe development in terms of discrete ‘phases’, in practice it was important to link activities and associated stakeholders to provide continuity, for example inviting industry partners to join clinical stakeholders in design validation activities to best understand their needs and working context.

*Commercialisation:* of the RAIS system represented the biggest potential challenge in the translational pathway. Commercial uptake of the system is necessary to scale provision to a wider clinical audience and to support regulatory approval, both essential to sustain long-term use. Similarly, it is important to recognize that for the manufacturer, a system like RAIS represents an uncertain commercial prospect: the market is emergent rather than well established and the system must necessarily be cost-effective to achieve success, prohibiting high profit margins to cover initial development costs [45]. However, operating as an academic research project provides an opportunity, and arguably a responsibility, to foster innovation in these areas of healthcare which may be otherwise neglected by de-risking the development process for all partners. Initial phases of commercialisation were undertaken as consultancy work by the manufacturer within the research project, supporting the design to manufacture process and compilation of regulatory documentation. This allowed independent development to ensure a focus on addressing clinical need, without commercial bias, but then provided a natural point to form a license with the manufacturer; the system design had matured and clinical viability had been demonstrated in cadaveric studies such that the commercial proposition was evident. Nevertheless, it is invaluable to identify a commercial partner who understands the humanitarian ethos of developing technology for ‘global health’, since even with risk mitigation it is likely to require an element of altruism to new technology such as RAIS until it becomes more established. This encompasses both the need for cost-effective systems and after-sales support infrastructure to enable maintenance and repair as required.

*Clinical Evaluation:* represented the final phase in the transfer of ‘ownership’ of the RAIS system, from the design team to clinical stakeholders and their wider community. This supports the project goals of achieving broader clinical use of the RAIS system which can be sustained, and grown, beyond the scope of an inherently time-limited research project. Thus, it was essential to ensure that RAIS is independently commercially-available and supported by clinical champions working locally within rural areas of India. In this respect, the importance of the clinical stakeholders cannot be overstated; from their investment in development of the system they have a detailed understanding of its operation and clinical potential. This has catalyzed activity in which they independently established clinical evaluation of the system together with training and a registry of use. Furthermore, they have received recognition of these activities with professional surgical societies (e.g. Association of Rural Surgeons of India) to foster broader clinical use, and ultimately patient benefit.

### A. Recommendations for Innovation

Through this translational work, a series of recommendations have been developed to guide others working on surgical device innovation for low resource settings:

• Embed Participatory Design from the selection of development methods to their implementation
• Establish a diverse group of stakeholders using existing clinical/research networks
• Promote regular communication with stakeholders
• Employ iterative design processes to support regular stakeholder interaction
• Engage an industry partner at an early stage
• Develop stakeholders into clinical champions to support long-term translation
• Link with wider clinical infrastructure to support e.g. training, mentorship, professional governance and certified equipment repairs

## V. Conclusion

Using a context-specific development pathway and participatory design principles was crucial in this project, particularly close and early engagement of stakeholders. This collaborative approach has enabled effective development and translation of RAIS into a surgical instrument used by an expanding team of surgeons in resource-scarce regions of India; realising the ambition of moving from clinical need to clinical use.

## Appendices

Part A provides supporting photo and video media of the RAIS system during clinical validation in rural surgical sites. Part B presents the participant information and questionnaire sheet for developmental validation (Methods B.1), conducted during system development. Part C presents the clinical validation questionnaires (Methods D).

## Supplementary Materials

Supplementary materials
